# A Bioinspired Approach to Mechanically Reinforce Collagen‐Rich Tissues Using Modularly Defined Stilbenoids

**DOI:** 10.1002/bip.70076

**Published:** 2026-01-05

**Authors:** Mahmoud Sayed Ahmed, Cheng‐Lei Wang, Shaonong Chen, Guido F. Pauli, Ana K. Bedran‐Russo

**Affiliations:** ^1^ Department of Oral Biology College of Dentistry, University of Illinois Chicago Chicago Illinois USA; ^2^ Department of Pharmaceutical Sciences Pharmacognosy Institute, Retzky College of Pharmacy, University of Illinois Chicago Chicago Illinois USA

**Keywords:** collagen cross‐linking, dentin biomodification, stilbenoids, structure–activity relationship (SAR), viscoelastic properties

## Abstract

Natural products, particularly plant‐derived compounds, have long inspired the development of novel dental biomaterials. This study explored the modularity and bioactivity of natural stilbenoids as dental tissue biomodulators, focusing on their ability to reinforce dentin mechanically through interactions with type I collagen. Mid‐coronal dentin specimens from human molars were demineralized and treated with 1% solutions of modularly defined stilbenoids (MoDS): a monomer (resveratrol), dimers (*ε*‐viniferin, ampelopsin A), and tetramers (vitisin A, vitisin B). Dynamic mechanical analysis (DMA) assessed viscoelastic properties (*E*′, *E*″, E*, tan *δ*) over 6 months, while Fourier‐transform infrared spectroscopy (FTIR) evaluated biochemical changes, and cell viability assays determined biocompatibility. Statistical analysis used ANOVA with post hoc tests (*α* = 0.05). Only oligomeric MoDS significantly enhanced dentin viscoelasticity (*p* < 0.001), with vitisin B showing the highest fold increase in E* modulus (17‐fold), followed by vitisin A and *ε*‐viniferin. FTIR confirmed permanent collagen modifications in oligomer‐treated groups, while resveratrol showed no mechanical effect. MoDS‐induced changes stabilized over time, and all compounds exhibited high biocompatibility, with vitisin B maintaining the highest cell viability. These results demonstrate that MoDS improve dentin mechanical properties and stability through structure‐dependent cross‐linking, highlighting their potential for durable, bioinspired dental restorations.

## Graphical abstract

A schematic diagram of dentin biomodification using modularly defined stilbenoids (MoDS). Stilbenoids interact with type I collagen at multiple hierarchical levels, inducing intermolecular and inter‐microfibrillar cross‐linking that enhances dentin viscoelastic properties, structural stability, and biocompatibility.

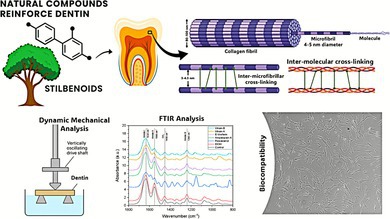

## 1 Introduction

The durability and success of dental restorations depend significantly on the stability of the dentin‐adhesive interface. However, this interface is vulnerable due to the hydrolytic breakdown of the resin matrix and the enzymatic degradation of type I collagen fibrils within the hybrid layer [1]. To address these challenges, various strategies have been explored to enhance adhesion to dentin. Dentin biomodification [2] is one of them: it utilizes a non‐enzymatic framework and involves a complex interplay between bioactive agents and the diverse extracellular components of the dentin matrix. The collective aim is to strengthen the dentin structure by enhancing dentin matrix stability and thereby enhancing its biomechanical features as well as overall biostability [2, 3].

Natural products, including those from plants, have long inspired the development of novel biomaterials [2]. Among them are phenolic compounds, particularly proanthocyanidins (PACs), which are oligomeric catechin derivatives that have recently been extensively studied in the dental field as collagen biomodulators. Their ability to reduce collagen solubilization, enhance dentin matrix stiffness, lower surface hydrophilicity, and decrease resin‐dentin interfacial permeability has positioned them as promising agents for enhancing the long‐term durability of adhesive interfaces [2, 4, 5]. One striking property of PACs is their building pattern as modular oligomeric plant phenols (MOPPs). The mechanism of action and potency of PACs depend on key structural features, such as the molecular size [6], the exact constitution of the catechin‐type building blocks [6, 7, 8], as well as the linkage type and degree of polymerization (DP) [6, 7, 9]. Plant stilbenoids represent another important class of MOPPs, which are structurally diverse but have yet to be explored systematically for dental biological endpoints.

In general, Nature employs modularity commonly when biosynthesizing essential macromolecules: examples are collagen and other proteins from amino acids, DNA/RNA from nucleotides, and polysaccharides/lectins from sugars. Vascular plants employ other, relatively simple building blocks to biosynthesize structurally highly diverse frameworks. Our recent recognition of the structure–activity relationships (SARs) that underlie PAC dentin bioactivity [7] has proven that biosynthetic modularity can infer specificity to biological interaction and/or recognition mechanisms, rather than being an expression of randomness and/or biological uniformity. The modular definition and phenolic character of stilbenoids inspired the present investigation towards their suitability as biomaterials that might help overcome the above biostability challenges in restorative and regenerative dentistry.

Despite growing interest in plant‐based phenolic oligomers and polymers such as stilbenoid, a critical knowledge gap remains regarding the mechanisms by which these compounds of varying molecular sizes and stereochemical architectures interact with type I collagen in dentin. Specifically, it is unknown whether oligomeric stilbenoids, comparable to PAC oligomers, exhibit a superior biomodification potential relative to their monomeric precursors. Particular knowledge gaps concern how their DP influences collagen mechanics, cross‐linking behavior, and long‐term dentin matrix stability. Therefore, a systematic comparison of monomeric, dimeric, and tetrameric stilbenoids can provide new rationales for a biological and chemical framework to identify key structural features that govern their dentin‐modifying capacity.

**FIGURE 1:**
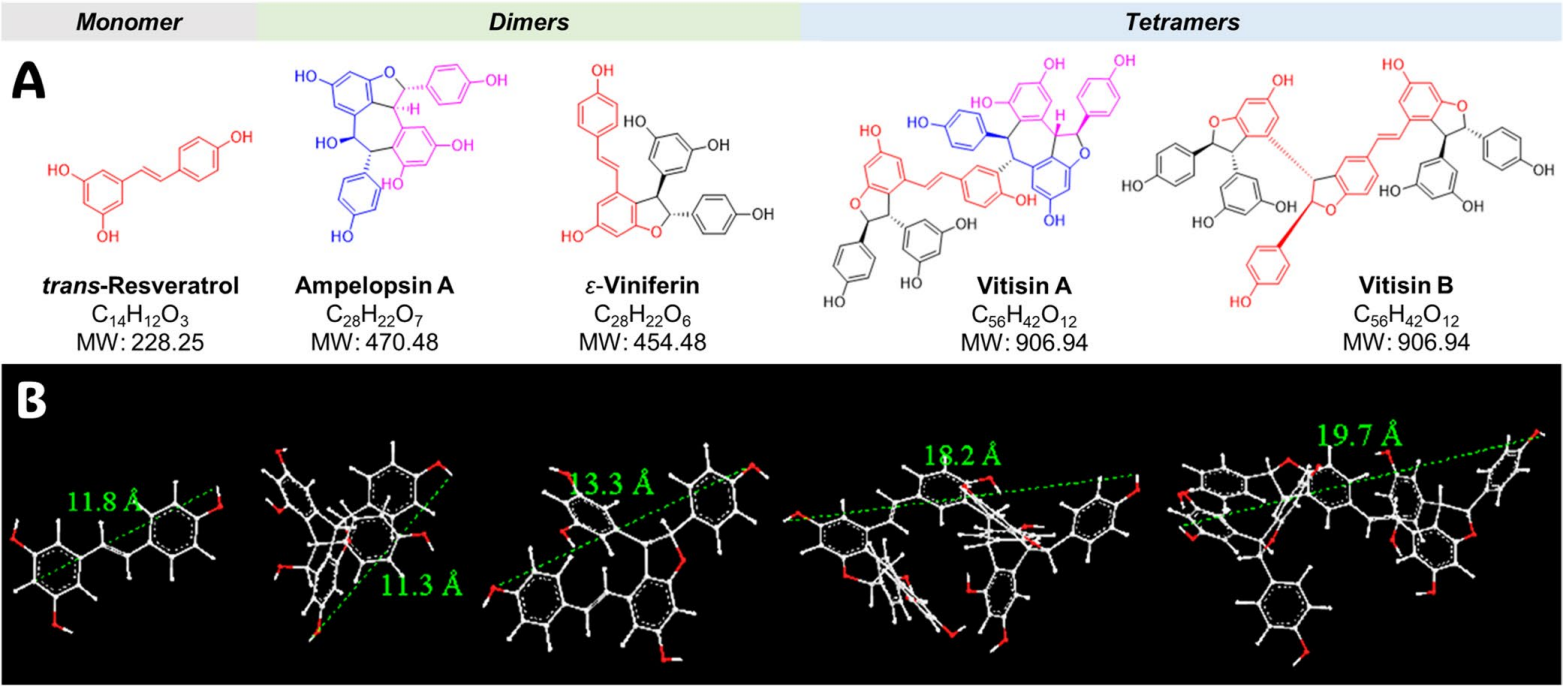
(A) Two‐dimensional chemical structures, formulas and molecular weight (MW) of the selected modularly defined stilbenoids (MoDs). The chemical structures highlight the modularity and structural relationships among the five MoDs (resveratrol, ɛ‐viniferin, ampelopsin A, vitisin A, and vitisin B). (B) Three‐dimensional models for MoDs were performed using Chem3D Pro of ChemDraw package (version 23.1.2, PerkinElmer, Waltham, MA). The distance between two hydrogen atoms was determined with the MM2 function during the molecular dynamics calculation for each molecule in angstroms (Å) to estimate molecular size.

Introduced by Gorham in 1980, the term “stilbenoids” refers to hydroxylated derivatives of stilbenes [10, 11, 12, 13]. Chemically, stilbenoids are phenolic compounds characterized by a structural skeleton comprising two benzene rings connected by an ethylene bridge [11] (Figure 1). Stilbenoids occur predominantly in plants of the *Gnetaceae*, *Pinaceae*, *Cyperaceae*, *Fabaceae*, *Dipterocarpaceae*, and *Vitaceae* families. Stilbenoids from the *Vitaceae*, such as those from *Vitis* species, have attracted increasing scientific interest due to their structural diversity and growing evidence of a variety of bioactivities [14, 15, 16, 17, 18].

Importantly, stilbenoids constitute MOPPs that share certain molecular characteristics (e.g., monomer size, phenolic rings) with PACs, yet are distinct by several other means (e.g., different linkage types, extended constitutional and stereochemical possibilities). Given the promising bioactive properties of MOPPs, this study investigated the role of key structural variations and molecular size of stilbenoids as potential biopolymers for dentin biomodulation. To do so, five modularly defined stilbenoids (MoDS) were purified from their natural source to examine their effects on the biomechanical and biochemical properties of the dentin extracellular matrix (ECM) in detail. We also evaluated their biocompatibility with dental pulp cells.

## 2 Materials and Methods

### 2.1 Stilbenoid Extraction and Isolation

The methanol extract of *Vitis labrusca* root bark (75 g) was partitioned between methyl *tert*‐butyl ether (MtBE) and water, yielding 38 g of the MtBE fraction. This fraction (36 g) was subjected to MCI gel chromatography using a nonlinear stepwise gradient elution with 20%, 30%, 50%, 70%, 80%, and 100% (aqueous) methanol, affording six fractions (M1–M6). Fractions M3–M5 contained the enriched MoDS. Fraction M3 was further separated by centrifugal partition chromatography (CPC) using a biphasic solvent system composed of *n*‐hexane/ethyl acetate/methanol/water (2:5:2:5, v/v) in descending mode at a flow rate of 10 mL/min and 3000 rpm, resulting in the isolation of ampelopsin A (765 mg). Fraction M4 was separated by CPC using a *n*‐hexane/ethyl acetate/methanol/water system (1:2:1:2, v/v) under the same operational conditions. Based on TLC analysis, the resulting CPC fractions were pooled into four subfractions (M4C1–M4C4). Subfraction M4C2 was purified on a Toyopearl HW‐40F column using 90% aqueous methanol to afford the major tetramer vitisin A (2441 mg). Subfraction M4C4 was subjected to Biotage RP18 flash chromatography with a gradient of 30%–70% aqueous methanol, yielding resveratrol (160 mg), *ε*‐viniferin (220 mg), and vitisin B (1790 mg). The structures of the five chemically defined stilbenoids are shown in Figure 1. The stilbenoids used in this study were purified by repeated, orthogonal chromatography, and their purities were verified by the 100% qHNMR method. The purities were as follows: vitisin A (84.8%), vitisin B (97.0%), *ε*‐viniferin (98.1%), ampelopsin A (98.5%), and trans‐resveratrol (98.2%). The qHNMR spectra (Figures S1–S5) and evaluation measures (Table S1) are provided in the Supporting Information to confirm both their identity and purity.

These four study compounds were selected as they are major stilbenoid constituents in the dentin‐bioactive roots of *V. labrusca* . Importantly, they represent different degrees and modes of oligomerization. Resveratrol is a monomer; *ε*‐viniferin and ampelopsin A are dimers formed through distinct coupling patterns. The vitisins A and vitisin B are tetramers. Collectively, these compounds form a structurally diverse set suitable for foundational SARs that support future investigations into stilbenoid‐mediated biomodification of dentin.

### 2.2 Specimen Preparation and Treatment

Extracted human sound molars were used to obtain dentin specimens (IRB no. 2023‐0717). Teeth were sectioned into mid‐coronal dentin standardized specimens (0.5 mm (thickness) × 1.7 mm (width) × 7.0 mm (length)). Specimens were demineralized in 10% phosphoric acid (Ricca Chemical Company, Arlington, TX, USA) for 5 h under constant agitation at room temperature. To ensure consistent positioning throughout the experimental procedures, a small dimple was created on one edge of each specimen using a diamond bur (#835, Brasseler USA Dental, Savannah, GA, USA), as previously described [19].

Based on our previous report and preliminary data [20, 21], the dentin was then treated with a 1% (w/v) concentration of each experimental compound prepared in a 70:30 solution of HEPES buffer (20 mM, pH 7)/ethanol (200 proof). For resveratrol treatment, the compound was dissolved in neat ethanol (96%) at the same final concentration. Two control groups were included: one via specimen immersion in 70% HEPES/30% ethanol and another treated with 96% ethanol (200 proof), both under otherwise identical experimental conditions. Each specimen was immersed in 100 μL of the respective solution for 1 h at room temperature in the absence of light. Following treatment, specimens were thoroughly rinsed three times with ultrapure water.

### 2.3 Dynamic Mechanical Analysis (DMA)

DMA was used to evaluate the viscoelastic properties of the dentin ECM. A strain‐sweep protocol was selected over a frequency‐sweep because it enables characterization of the linear viscoelastic region and sensitively reflects cross‐linking–induced changes in the dentin matrix under increasing deformation amplitudes.

Dentin specimens were submerged in ultrapure water and analyzed at room temperature using a three‐point bending submersion clamp equipped with a vertically oscillating drive shaft (Q800 DMA, TA Instruments, New Castle, DE, USA). The testing parameters were set as follows: frequency of 1 Hz, amplitude ranging from 1 to 100 μm, and a preload force of 0.01 N. This strain sweep method allowed for the characterization of dentin's viscoelastic response under dynamic loading conditions across varying strain amplitudes, maintaining a constant frequency and temperature throughout the experiment [19].

The viscoelastic behavior of the dentin ECM was quantified by measuring the storage modulus (*E*′), loss modulus (*E*″), and complex modulus (E*), which represent the elastic, viscous, and total viscoelastic response of the material, respectively. The damping capacity (tan *δ*), a ratio of the loss modulus to the storage modulus (tan *δ* = *E*″/*E*′), reflects the material's energy dissipation characteristics [19].

Pre‐screening was done on all demineralized dentin specimens to identify those with complex modulus baseline values between 6 and 10 MPa. Approximately 30% of the prepared dentin beams were excluded during pre‐screening because their initial complex modulus (*E*) did not fall within the defined range for demineralized dentin. The experimental groups were then assigned these specimens. Specimens (*n* = 8) were incubated in simulated body fluid (SBF) (5 mmol/L HEPES, 2.5 mmol/L CaCl_2_, 0.05 mmol/L ZnCl_2_, and 0.3 mmol/L NaN_3_, pH 7.4) at 37°C, and measurements were performed at four time points (immediate, 2 weeks, 3 months, and 6 months post‐treatment).

### 2.4 Physicochemical Analysis of the Dentin Extracellular Matrix

The biochemical characterization of functional groups within the dentin ECM was conducted using Fourier‐transform infrared spectroscopy (FTIR) on a Nicolet 6700 spectrometer (Thermo Fisher Scientific), equipped with an attenuated total reflectance (ATR) apparatus featuring a single‐reflection diamond crystal. Dentin specimens (*n* = 5) were demineralized and treated as described in Section 2. Spectral data were collected in the range of 4500–600 cm^−1^, with 128 scans accumulated per sample. Prior to each analysis, background transmission spectra were acquired without a specimen on the ATR crystal to facilitate absorbance spectrum computation. The specimens (*n* = 5) were then pressed onto the ATR plate surface for spectral acquisition. Specimens were incubated and measured at different time points as described in Section 3.

Post acquisition, spectra underwent a 14‐point interpolated baseline subtraction and were normalized for the mean values of all datasets. Analysis of the collagen secondary structure was performed by correlating the frequencies of amide bands with patterns of hydrogen bonding that represent the backbone of collagen polypeptide chains, including *α*‐helices and *β*‐sheets [18, 22]. Specific attention was given to the dominant infrared absorption bands of type I collagen: amide I (C=O stretching at ~1630 cm^−1^), amide II (N—H bending and C—N stretching at ~1550 cm^−1^), amide III (C—N stretching and N—H bending at ~1240 cm^−1^), and the CH_2_ wagging vibrations at ~1450 cm^−1^. Band intensities and area integrals of these spectral features were fitted and calculated to assess structural variations in collagen cross‐linking and triple‐helix conformation. FTIR‐derived indices were calculated as ratios between the amide II and amide III bands versus the CH_2_ scissoring band (amide II/CH_2_ and amide III/CH_2_). Spectral processing and visualization were performed using OMNIC Spectra (Thermo Fisher Scientific) and Origin Pro 8 (OriginLab, Northampton, MA, USA).

### 2.5 Biocompatibility and Cytotoxicity Analysis

Human dental pulp‐derived mesenchymal stem cells (DPSCs) were cultured in a modified Minimum Essential Media (α‐MEM, Corning, 15‐012‐CV, USA) supplemented with 20% Fetal bovine serum (FBS, Corning, 35‐010‐CV, USA), 1% l‐glutamine, and 1% antibiotic/antimycotic solution (Corning, 30‐009‐CI, USA). DPSCs were seeded onto 96‐well plates at a concentration of 1 × 10^4^ cells/well at 37°C in 5% CO_2_. After culturing for 24 h to allow cell adhesion, the culture medium was replaced with the experimental medium. The purified stilbenoids were dissolved in culture media to obtain different concentrations (1, 10, 100 μg/mL, *n* = 6). These concentrations were selected based on established cytotoxicity testing ranges for natural polymeric phenols [20, 23], enabling assessment of low, intermediate, and high challenge doses. While the lower concentrations reflect ranges typical for biomodulator biocompatibility studies, 100 μg/mL is an intentionally escalated exposure level to evaluate cellular tolerance beyond expected clinical relevance. Cells cultured without MoDS served as a blank control. Cell proliferation was measured using PrestoBlue Cell Viability Reagent (Invitrogen, A13262, Thermo‐Fisher Scientific Inc., USA) following the protocol guidelines specified by the manufacturer. Absorbance was measured using a microplate reader (BioTek Synergy HTX plate reader), set at 540 nm for fluorescence excitation, acquiring readings after 1, 3, and 5 days.

### 2.6 Statistical Analysis

Data were subjected to normality and homogeneity tests using Levene's test with a significance level set at *p* < 0.05. Data were statistically analyzed using a general linear model through repeated‐measures ANOVA/two‐way ANOVA, with experimental compounds and time points as independent variables. Post hoc comparisons were conducted using Tukey's or Games Howell tests (*α* = 0.05) (SPSS v.25, SPSS Inc., Chicago, IL, USA).

## 3 Results

### 3.1 Dynamic Mechanical Analysis

**FIGURE 2:**
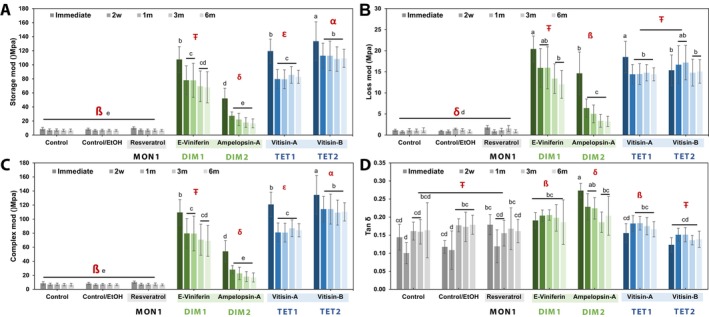
Results of the dynamic mechanical analysis of MoDS‐modified dentin matrices at different time points (*n* = 8). The graphs show means and standard deviation bars of (A) Storage modulus, (B) Loss modulus, (C) complex modulus, and (D) tan *δ* of MoDS‐modified dentin as a function of their distinct degrees of polymerization (DP) and specific compound/structure. Different symbols (ß, Ŧ) indicate significance between treatments (*p* < 0.05). Different lowercase roman letters (a, b) indicate significance between time points (*p* < 0.05).

The effects of biomodification of dentin ECM with stilbenoids on the viscoelastic properties are presented in Figure 2. Significant interactions were observed between the treatment groups and time points (*p* < 0.001) for all viscoelastic parameters, including the complex modulus (E*), storage modulus (*E*′), loss modulus (*E*″), and damping capacity (tan *δ*). All compounds significantly enhance the dentin matrix biomechanical properties, as indicated by increased *E*′ and *E*′′ values, except for resveratrol (monomer), which showed no significant effect (*p* = 1.00).

Among the tested compounds, tetrameric stilbenoids demonstrated the greatest impact immediately after treatment. Vitisin B and A treatments yielded the highest fold increases in E* modulus at 16.9 (from 8 to 133.5 MPa) and 15.4 (from 8.04 to 119.38 MPa), respectively. Dimeric stilbenoids followed in ranks, with *ε*‐viniferin and ampelopsin A producing fold increases of 13.72 (from 8.06 to 107.56 MPa) and 6.63 (from 8.05 to 52.07 MPa), respectively. In contrast, monomeric resveratrol and both control groups showed no measurable differences in mechanical properties across all timepoints (*p* = 1.00).

After 2 weeks, a significant decrease (*p* < 0.001) in all dentin moduli was noted: E* modulus values declined by ~16% for vitisin B, ~33% for vitisin A, ~30% for *ε*‐viniferin, and ~48% for ampelopsin A. Nevertheless, the mechanical properties (E*, *E*′, and *E*″) of all oligomeric stilbenoid‐treated groups remained stable from the 2‐week mark through 6 months except for the dimeric ampelopsin A showing the least stability, with a gradual but non‐significant decrease in E* observed at 1 m (*p* = 0.99), 3 m (*p* = 0.49), and 6 m (*p* = 0.25).

Changes in the *E*″ modulus over time were mirrored by variations in damping capacity values at corresponding intervals. Interestingly, the tetramer vitisin B exhibited damping capacity values comparable to the control groups, with no significant differences (*p* = 0.99) observed between them. However, vitisin B demonstrated greater stability over time, maintaining its mechanical performance more consistently throughout the evaluation period.

### 3.2 Biochemical Analysis of Extracellular Dentin Matrix

**FIGURE 3:**
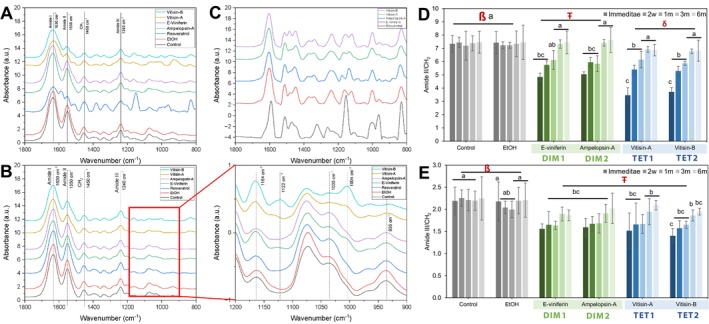
Biochemical characterization and structural modifications of stilbenoid‐modified dentin matrices at different time points (*n* = 5). FT‐IR spectra of unmodified (control) and dentin matrix biomodified by MoDS (A) Immediately after biomodification, and (B) 2 weeks after biomodification. Peaks assigned to dentin ECM are represented by the dashed lines (Amide I at 1630 cm^−1^, Amide II at 1550 cm^−1^, amide III at 1240 cm^−1^, and CH_2_ scissoring at 1450 cm^−1^). Biomodified dentin matrices exhibited changes in the absorption bands (900‐1200 cm^−1^) after biomodification (B, right). (C) Representative FT‐IR spectra of the MoDS alone, collected to identify the characteristic absorption bands and to aid interpretation of material‐specific contributions to the dentin spectra. The graphs show means and standard deviation bars of the ratios calculated from the intensities and areas under the assigned peaks of (D) amide II versus CH_2_ (1550/1450 cm^−1^) and (E) amide III versus CH_2_ scissoring (1240/1450 cm^−1^). Different symbols (ß, Ŧ) indicate significance between treatments (*p* < 0.05). Different lowercase roman letters (a, b) indicate significance between timepoints (*p* < 0.05).

Representative ATR‐FTIR spectra of the dentin ECM displaying characteristic type I collagen peaks are shown in Figure 3. This includes the amide I (~1630 cm^−1^), amide II (~1550 cm^−1^), CH_2_ scissoring (~1450 cm^−1^), and amide III (~1240 cm^−1^) bands. Treated specimens exhibited spectral modifications absent in untreated controls. Notable observations included decreased intensity and broadening of amide bands, minor spectral shifts in the amide I, II, and CH_2_ bands, and the emergence of new bands in the treated groups. Specifically, the vitisin B spectra revealed a new band at ~1122 and ~1004 cm^−1^, heightened intensity at ~1164 cm^−1^, and a loss of bands at ~1035 and ~935 cm^−1^. The *ε*‐viniferin‐treated matrices displayed a spectral profile similar to vitisin B, whereas ampelopsin A and vitisin A exhibited comparable patterns.

The FTIR spectra of resveratrol‐treated samples exhibited numerous intense absorption bands attributable to the compound itself [24], as could be expected from the treatment. These bands overlapped with collagen bands, as confirmed by comparison with stilbenoids‐only spectra in Figure 3C. As a result, collagen structural changes could not be reliably detected, and Amide II/CH_2_ and Amide III/CH_2_ ratios were not calculated for the resveratrol group.

In other treated groups, the Amide II/CH_2_ ratio showed a significant reduction immediately post‐treatment (*p* < 0.001), in particular for the vitisin B and A groups, followed by a gradual increase over subsequent time points. The Amide III/CH_2_ ratio mirrored this pattern. These trends suggest a partial reversal of the initial MoDS‐induced collagen biomodification. However, the persistence of newly formed bands in the MoDS‐modified ECM spectra Figure 3E suggests that a permanent chemical shift occurred in the stilbenoid‐dentin complex.

### 3.3 Biocompatibility and Cytotoxicity

**FIGURE 4:**
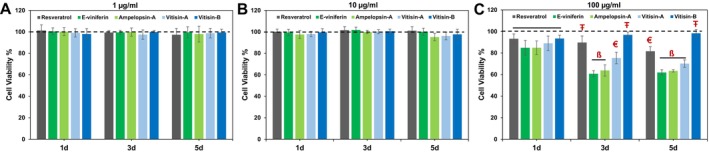
Proliferation of DPSCs after exposure to different MoDS treatments at different timepoints (*n* = 6). (A) Direct treatment of DPSCs cultured with 1 μg/mL refined mixture; (B) Direct treatment of DPSCs cultured with 10 μg/mL refined mixture; (C) Direct treatment of DPSCs cultured with 100 μg/mL refined mixture. *n* = 6 replicates per culture condition. The two dash lines represent the cytotoxic effect in accordance with ISO 10993–2005. Different symbols (ß, Ŧ) indicate significance between treatments within each time point (*p* < 0.05).

Results of cell proliferation and viability assays are shown in Figure 4. Across all experimental groups and time points, the investigated stilbenoids demonstrated good biocompatibility. At concentrations of 1 and 10 μg/mL, high levels of cell proliferation were observed over 1, 3, and 5 days of incubation, with no significant differences (*p* < 0.001) among groups. However, at a concentration of 100 μg/mL, a marked decline in cell viability was observed in the resveratrol, *ε*‐viniferin, ampelopsin A, and vitisin A groups, confirmed after 5 days of exposure. Markedly, the vitisin B group maintained significantly high cell viability across all concentrations and time points (*p* < 0.001), indicating superior cytocompatibility compared to the other compounds.

## 4 Discussion

MOPPs illustrate the principle of modularity in natural product biosynthesis through their assembly from phenolic building blocks, yielding motifs with varied stereochemistry and bonding patterns. These modular yet variable construction principles generate substantial structural diversity and affect how MOPPs interact with collagen. Stilbenoids are one major class of MOPPs that are characterized by a C_6_—C_2_—C_6_ monomeric backbone in which two aromatic rings are connected via an ethylene bridge [25, 26]. The position and number of hydroxyl and/or methoxy groups, especially on ring B, influence bioactivity [26].

The diversity of modularity is evident when comparing the four oligomeric MoDs investigated, *ε*‐viniferin, ampelopsin A, and the vitisins A and B. Although both *ε*‐viniferin and ampelopsin A are resveratrol‐derived dimers featuring a benzofuran moiety, they are structurally distinct. Their dimerization involves phenolic oxidative coupling, while the formation of ampelopsin A involved further oxidative rearrangement, resulting in a seven‐membered ring. This structural feature yields a significantly more rigid and compact 3D topology compared to *ε*‐viniferin. Being tetramers, the vitisins A and B represent higher‐order assemblies. Vitisin A integrates *ε*‐viniferin and ampelopsin A units, while vitisin B is composed entirely of two *ε*‐viniferin modules. Similarly to PACs, higher‐oligomeric stilbenoids structures not only have an increased molecular size and molecular weight but also gain spatial reach, which impacts how they can mediate cross‐linking interactions at the intermolecular and/or inter‐microfibrillar level. In contrast, monomers such as resveratrol cannot achieve these types of interactions due to their smaller size and structural limitations, which explains the observed essential lack of dentin bioactivity. The distinct modular features across the panel of tested oligomers explain their different affinities and modulatory effects on the biomechanics of collagen‐based substrates, which can be discussed in more detail as follows:

For a better perspective of such a discussion, it shall be pointed out that type I collagen, the principal organic component of dentin, is a heterotrimeric molecule composed of two *α*1 and one *α*2 chains, each comprising three domains: the NH_2_‐terminal non‐triple helical domain (N‐telopeptide), the central triple helix, and the COOH‐terminal non‐triple helical domain (C‐telopeptide) [27]. In dentin, the collagen molecules self‐assemble into fibrils, which further organize into complex hierarchical structures that are essential to the tissue's viscoelastic behavior and toughness [28, 29]. Post‐translational modifications at various hierarchical levels, including intramolecular, intermolecular, and inter‐microfibrillar cross‐linking, enable both biostability and mechanical integrity [27, 30, 31].

Our findings reveal that both the initial and sustained dentin bioactivity elicited by MoDS closely depend on the compounds' distinct structural features, in particular their molecular shape, including stereochemistry and DP. This emphasizes the critical role of the unique modularity of MoDS and highlights its relevance for their development as interventional and targeted biomaterials.

The cross‐linking potential of MoDS oligomers can primarily be attributed to the presence and spatial orientation of phenolic hydroxyl groups and multiple aromatic rings [32]. Phenolic hydroxyl groups act as effective hydrogen donors, enabling the formation of hydrogen bonds with carbonyl groups in collagen, which serve as hydrogen receptors [33]. Conversely, the free amino groups in collagen can function as hydrogen donors, forming complementary hydrogen bonds with the phenolic hydroxyl groups in the cross‐linkers acting as hydrogen receptors [34]. In addition to hydrogen bonding, hydrophobic interactions play a crucial role. The aromatic rings of MoDS are available to interact with the hydrophobic side chains of amino acids in collagen, and their exact spatial position depends largely on the overall constitution and stereochemistry of each molecule. The dual interaction mechanism of H‐binding and hydrophobic pi‐stacking has been described since the early 1980s and has widely been accepted as the “glove‐hand” model [35]. According to this theory, complex phenolic natural products initially associate with protein surfaces via hydrophobic interactions, subsequently penetrate hydrophobic pockets within the protein, and eventually form multiple hydrogen bonds that provide a more robust and multipoint anchoring mechanism.

**FIGURE 5:**
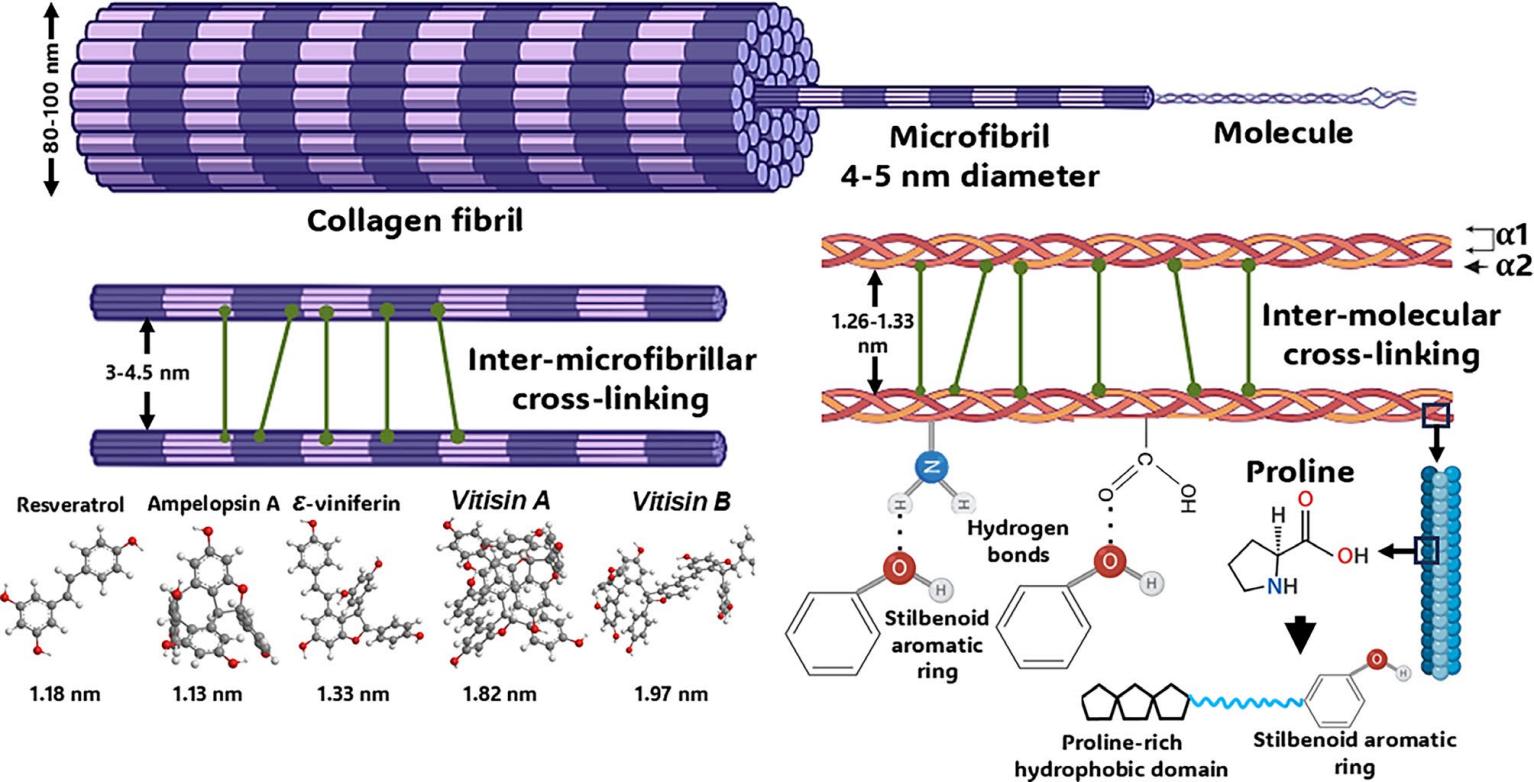
Hierarchical structure of collagen fibrils and potential mechanisms of dentin biomodification guidance. Each collagen fibril is composed of smaller units called microfibrillar bundles, which have a diameter of approximately 10–25 nm. The microfibrils are structures with a dimension of 4–5 nm and are separated by 3–4.5 nm. Thus, tetrameric vitisin A and vitisin B are expected to mediate inter‐microfibrillar cross‐linking. The mechanical characteristics may be greatly impacted by the inter‐microfibrillar cross‐links. Five 1D staggered, rope‐like, right‐hand twisted collagen molecules make up each microfibril. The 1.26–1.33 nm separation of collagen molecules permits the intermolecular cross‐linking of monomeric resveratrol, dimeric ampelopsin A, and *ε*‐viniferin.

Derived from resveratrol oligomerization, MoDS display distinct interaction behaviors owing to their well‐defined yet diverse molecular architectures. Factors such as the number, orientation, and position of hydroxyl groups are likely to influence their cross‐linking effects across the highly organized hierarchical levels of collagen, including the fibrillar, sub‐fibrillar, and micro‐fibrillar structures. The exact chemical structure of each individual stilbenoid possesses key features beyond the presence of hydroxyl groups, including the olefinic bond characteristic: while one of the unsaturations of the resveratrol precursor is still present in *ε*‐viniferin, this is no longer the case in ampelopsin A; similarly, unsaturation can vary in higher order MoDS. Additionally, flexible C—O—C ether linkage, such as in *ε*‐viniferin, may enhance chemical interactions with dentin and may confer greater conformational adaptability and spatial fit to MoDS, thereby exposing more phenolic groups for interaction with collagen. Moreover, the multiple aromatic rings in stilbenoids can enhance the stability of interactions via π–π stacking with proline‐rich regions in collagen, creating additional non‐covalent stability and hydrophobicity (Figure 5).

DMA was employed to evaluate shifts in the viscoelastic behavior of dentin ECM. This revealed mechanical performance parameters such as storage, loss, and complex moduli. The damping capacity (tan *δ*) provided deeper insight into molecular mobility, collagen cross‐linking, and ECM rearrangements during mechanical loading [36, 37]. All oligomeric MoDS significantly enhanced the dentin matrix mechanical properties as reflected by increases in storage (*E*′), loss (*E*″), and complex moduli (E*). The increased damping capacity (tan *δ*) indicates the presence of high‐density hydrogen bonding interactions, likely between MoDS hydroxyl groups and collagen amide carbonyls [38, 39]. FTIR analysis supports these conclusions as it revealed a significant reduction in the amide II/CH_2_ ratio in treated specimens, indicating enhanced cross‐linking activity. Repeated mechanical loading is known to further influence tissue viscosity through reversible protein‐solvent hydrogen bond dynamics [36].

Among the compounds studied, *ε*‐viniferin and the vitisins A and B exhibited the most substantial mechanical reinforcement, showing a 14‐ to 17‐fold increase in storage modulus, whereas ampelopsin A showed a more modest yet still significant ~6‐fold enhancement. These differences can be projected onto variations in their molecular structure and flexibility. Although ampelopsin A contains one more hydroxyl group than *ε*‐viniferin (six vs. five, respectively), the lower bioactivity of ampelopsin A may result from a more rigid stereochemical configuration, whereas *ε*‐viniferin's olefinic bond provides greater structural flexibility and accessibility.

As could be expected from prior studies with PACs as another group of MOPPs, molecular size also plays a crucial role in the dentin bioactivity of MoDS. The molecular size of each compound was estimated by measuring the longest distance between atoms in each molecule (Figure 1). While dynamic light scattering analysis would be necessary to confirm the exact size distribution of these compounds [40]. The following values can be considered suitable estimates: resveratrol 1.18 nm, ampelopsin A 1.13 nm, *ε*‐viniferin 1.33 nm, vitisin A 1.82 nm, and vitisin B 1.97 nm; all fall within a size range that readily permits diffusion into collagen fibrils. These sizes are also compatible with both intrafibrillar and interfibrillar spaces, particularly when considering that collagen molecules are spaced 1.26–1.33 nm apart [41] and microfibrils 3–4.5 nm [42]. Furthermore, the molecular weights of the investigated compounds (resveratrol 228 Da, *ε*‐viniferin 454 Da, ampelopsin A 470 Da, vitisin A and B both 906 Da) confirm that all MoDS are well below the 6 kDa threshold known to facilitate efficient diffusion into the collagen matrix [43].

Although hydrogen bonds may be disrupted over time in aqueous environments such as SBF or artificial saliva [34], MoDS‐treated dentin maintained improved mechanical properties beyond the initial decline observed after 2 weeks. This suggests that many of the newly formed interactions remained stable within the matrix. In contrast, untreated dentin exhibited fluid‐like behavior after 6 months, likely due to collagen degradation by endogenous proteases [44, 45]. While widely reported as having a host of biological properties, which can also be interpreted as a classical IMP property [46]. Resveratrol—the monomeric precursor of MoDS—exhibited no biomechanical utility. Despite its small size and ease of diffusion, the monomeric precursors of MOPPS evidently lack the *multivalent* binding capabilities necessary for effective collagen reinforcement, a finding consistent with previous studies on the precursors of PACs such as catechin and epicatechin [6, 44]. However, FTIR analysis revealed numerous intense absorption bands attributable to resveratrol immediately after application, which could be interpreted as a sign of its potential ability to bind to the collagen surface. However, these spectral signatures disappeared after 2 weeks, suggesting that resveratrol may have been washed away over time due to weak and/or non‐permanent initial interactions.

Spectroscopic analysis further confirmed the observed MoDS‐induced biomodification (Figure 3). FTIR spectra of treated dentin revealed decreased amide band intensity, indicating conformational changes in the collagen triple helix and the formation of exogenous cross‐links [7, 19, 47] through increased hydrogen bonds and rearrangement of the existing collagen‐hydrogen bond pattern [5, 7]. The amide II absorption band is known to indicate the amount of —NH_2_ in collagen [48, 49]. A reduced amide II/CH_2_ ratio suggests transformation of free —NH_2_ groups into N—H bonds via hydrogen bonding [5, 50]. Alterations in the amide III band, linked to triple‐helix integrity, further confirm protein conformational rearrangement [50]. MoDS are structurally diverse [26, 51, 52] and the observed spectral differences across various MoDS treatments reflect their chemical diversity and indicate distinct dentin interaction mechanisms. The partial reversal of collagen modifications over time (starting at the 2 weeks time point) suggests that the long‐term stability of biomodification depends on the nature of the cross‐links formed. However, the persistence of new FTIR bands in all treated groups (notably except for resveratrol) confirms that MoDS treatment results in permanent chemical modifications of the dentin matrix.

Importantly, the MoDS demonstrated high biocompatibility, which is supported by their generally documented lack of toxicity. These effects are mediated through activation of pathways such as Nrf2, heme oxygenase‐1 (HO‐1), and *γ*‐glutamylcysteine synthetase (*γ*‐GCS), as well as the downregulation of proinflammatory cytokines like IL‐1β [51, 53], contributing to their overall favorable biological profile. Particularly, among all compounds tested, vitisin B emerged as the most biocompatible, maintaining significantly high cell viability even at the highest tested concentration of 100 μg/mL and highlighting its potential as the most promising candidate within the current test regimen for safe and effective dentin biomodification.

This study introduced structurally diverse MoDS, which vary in DP, inter‐stilbenoid linkages, and stereochemistry, as potentially clinically relevant dental biopolymers. The outcomes identified especially the oligomeric forms of MoDS, such as *ε*‐viniferin and the vitisins A and B, as potent dentin biomodulators. The distinct molecular features significantly influence the type and density of cross‐links formed with dentin collagen [2, 4, 6, 44, 54], which in turn affects the durability of biomodification, a key factor in the longevity of dental adhesive restorations [55].

## 5 Conclusion

The effectiveness of MoDS is strongly structure‐dependent, with DP and stereochemistry emerging as key SAR determinants. Higher‐order oligomers demonstrated enhanced mechanical reinforcement, with vitisin B showing the most favorable performance in both mechanical stability and biocompatibility. These effects were supported by IR‐spectroscopic evidence of stable chemical modifications, including the emergence of new absorbances. Collectively, our findings confirm that MoDS can engage collagen across multiple hierarchical levels through a combination of hydrogen bonding, hydrophobic interactions, and structural flexibility. This highlights their promise as dental tissue modulators and warrants their continued exploration as bio‐inspired, multifunctional interventional biomaterials.

## Funding

This study was supported by a grant from the National Institutes of Dental Craniofacial Research (NIDCR) [DE032547].

## Conflicts of Interest

The authors declare no conflicts of interest.

## Supporting information


**Data S1:** Supporting Information.

## Data Availability

The data that support the findings of this study are available from the corresponding author upon reasonable request.
