# Supplementary material for: A Bioinspired Approach to Mechanically Reinforce Collagen‐Rich Tissues Using Modularly Defined Stilbenoids
Source: Biopolymers. 2026 Jan 5;117(1):e70076. doi: 10.1002/bip.70076 (PMC12767555; doi:10.1002/bip.70076)

Title: A Bioinspired Approach to Mechanically Reinforce Collagen-Rich Tissues Using Modularly Defined Stilbenoids

Authors: Mahmoud Sayed Ahmed[1], Cheng-Lei Wang[2], Shaonong Chen[2], Guido F. Pauli[2], Ana K. Bedran-Russo[1*]

1. *Department of Oral Biology, College of Dentistry, University of Illinois Chicago, Chicago, IL, USA*
2. *Pharmacognosy Institute & Department of Pharmaceutical Sciences, Retzky College of Pharmacy, University of Illinois Chicago, Chicago, IL, USA*

Corresponding author:

Dr. Ana K Bedran-Russo, DDS, MS, PhD Professor

Distinguished Professor and Head, Department of Oral Biology

University of Illinois Chicago, College of Dentistry

801 S. Paulina Street, room 402E,

Chicago, IL 60612

Phone: 312-996-7515

Email: bedran@uic.edu

Supporting Information

Table S1. qHNMR purity of the five stilbenoids isolated from *Vitis labrusca* roots.

sample name	ORGANISM	MW	Purity by qHNMR 100% method
trans-resveratrol	Vitis Labrusca, roots	244	98.2%
ε-viniferin	Vitis Labrusca, roots	454	98.1%
ampelopsin A	Vitis Labrusca, roots	470	98.5%
vitisin A	Vitis Labrusca, roots	906	84.8%
vitisin B	Vitis Labrusca, roots	906	97.0%

Figure S1. ^{1}H NMR spectra of trans-resveratrol.

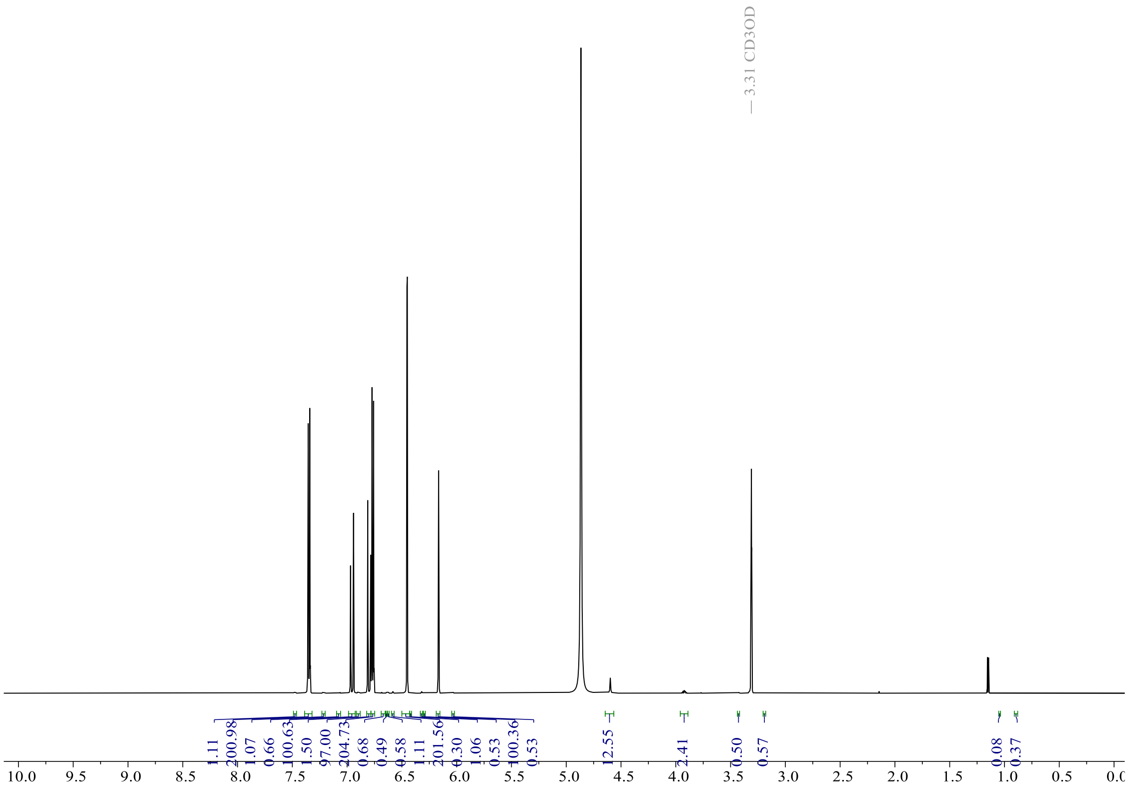

Figure S2. ^{1}H NMR spectra of *ε*-viniferin.

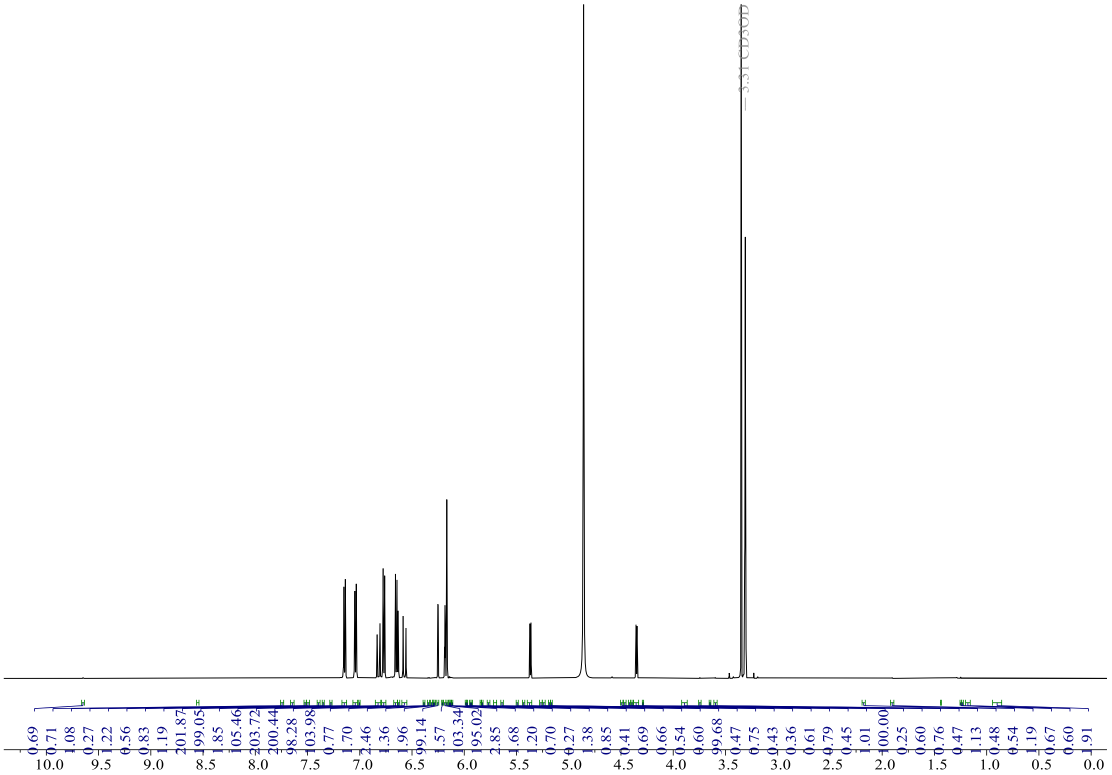

Figure S3. ^{1}H NMR spectra of ampelopsin A.

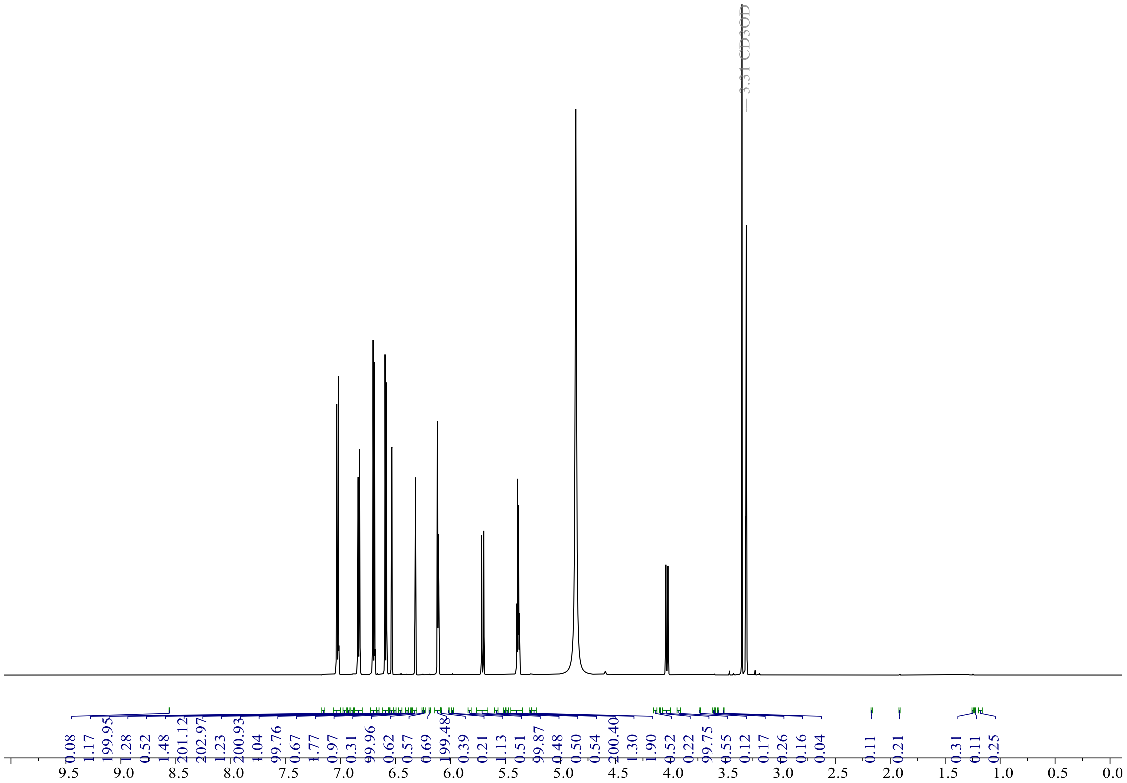

Figure S4. ^{1}H NMR spectra of vitisin A.

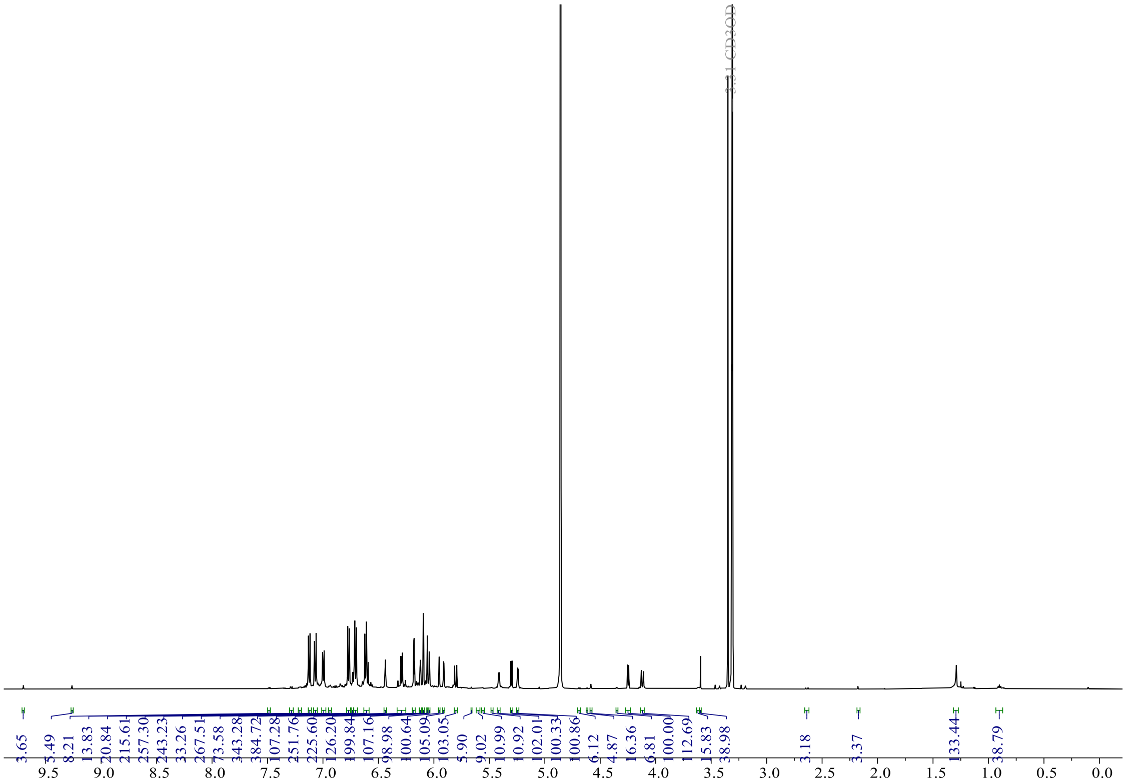

Figure S5. ^{1}H NMR spectra of vitisin B.

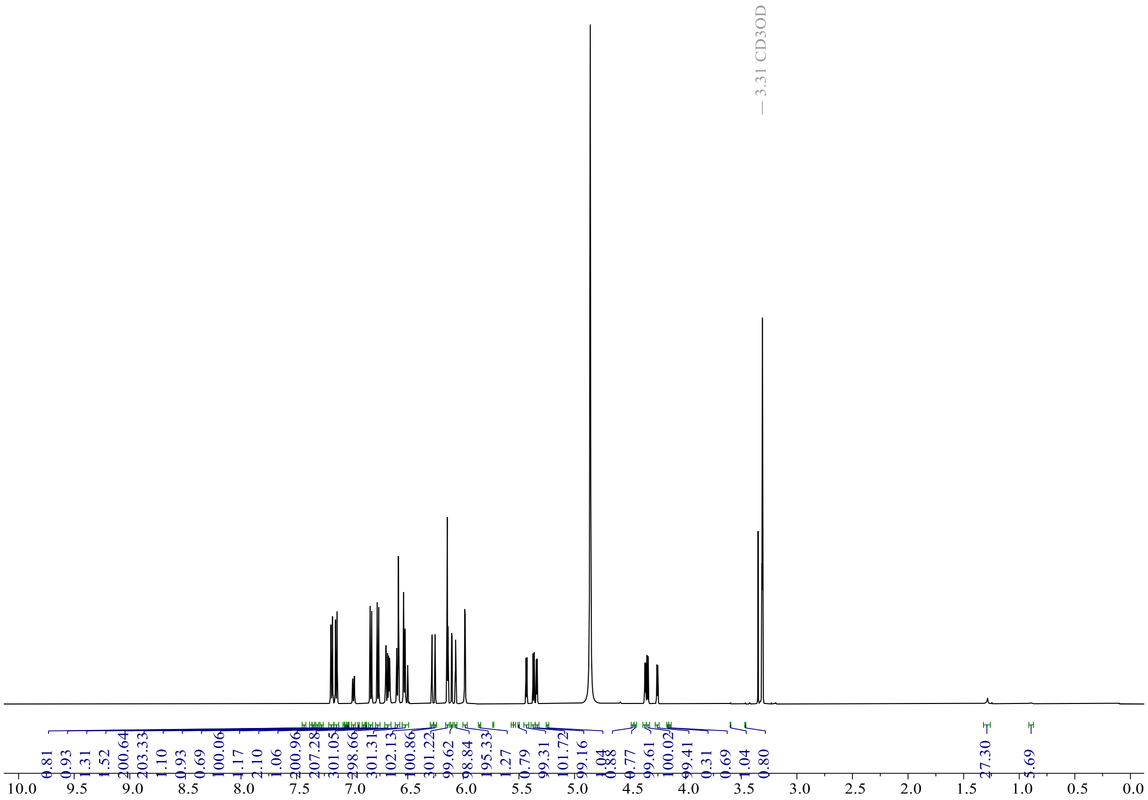

Supplement: Supplementary file 1 — Data S1: Supporting Information. [file BIP-117-e70076-s001.pdf]
